# FoxP3+ Cells and PNAd+ Tumour-Associated High Endothelial Venules: Synergistic Prognostic Markers in Oral Tongue Squamous Cell Carcinoma

**DOI:** 10.1007/s12105-026-01904-4

**Published:** 2026-03-26

**Authors:** Ibrahim Afolabi Abdulsalam, Kjersti Sellæg, Faith O. Benebo, Nancy C. Ojei, Sonja E. Steigen, Lars Uhlin-Hansen, Inger-Heidi Bjerkli, Anna M. Wirsing, Synnøve Norvoll Magnussen, Elin Hadler-Olsen

**Affiliations:** 1 Department of Medical Biology, Faculty of Health Sciences, UiT – The Arctic University of Norway, Tromsø, Norway; 2 Department of Community Medicine, UiT – The Arctic University of Norway, Tromsø, Norway; 3 Department of Clinical Pathology, University Hospital of North Norway, Tromsø, Norway; 4 The Public Dental Health Service Competence Centre of Northern Norway, Tromsø, Norway

**Keywords:** Oral tongue squamous cell carcinoma, Prognostic biomarkers, Tumour microenvironment, Tumour-associated high endothelial venules, Regulatory T cells, Competing risk analysis

## Abstract

**Purpose:**

Oral tongue squamous cell carcinoma (OTSCC) features significant immune cell infiltration. In head and neck SCC, peripheral node addressin (PNAd) + tumour-associated high endothelial venules (TA-HEVs) and CD163+ tumour-associated macrophages (TAMs) are associated with favourable and unfavourable prognoses, respectively, whereas the prognostic value of FoxP3+ cells is controversial. This national, multi-centre retrospective study evaluates the prognostic roles of these biomarkers individually and in combination in a homogeneous cohort of 126 treatment-naïve OTSCC patients diagnosed in Norway (2005–2009).

**Methodology:**

Immunohistochemistry on formalin-fixed, paraffin-embedded tissue assessed PNAd+ TA-HEVs, CD163+ TAMs, and FoxP3+ cell densities. Associations between scores and clinical/pathological variables were analysed using chi-square tests. Five-year disease-specific death (DSD) prediction was evaluated using cumulative incidence function estimation and Fine-Gray subdistribution hazard modelling to account for competing mortality.

**Results:**

In multivariable competing-risk analyses, high FoxP3⁺ cell density independently predicted increased five-year DSD (sHR = 5.40, 95% CI 1.92–15.17). PNAd demonstrated context-dependent prognostic effects when analysed with FoxP3: high PNAd/low FoxP3 tumours showed excellent prognosis, whereas low PNAd/high FoxP3 conferred highest risk (combined model: PNAd sHR 2.58, 95% CI 1.25–5.34; FoxP3 sHR 8.78, 95% CI 3.53–21.87). CD163⁺ TAM density was not associated with DSD. Combined biomarker assessment added incremental prognostic value beyond pTNM staging, with higher discrimination (C-index 0.727 → 0.784) and improved model fit (ΔAICc =  − 8.93; *p* < 0.0001).

**Conclusions:**

Higher FoxP3+ cell density independently predicted increased DSD, while PNAd showed context-dependent prognostic value. Combined biomarker assessment improved risk stratification beyond pTNM staging, supporting investigation of integrated immune profiling for risk stratification, pending external validation.

**Graphical Abstract:**

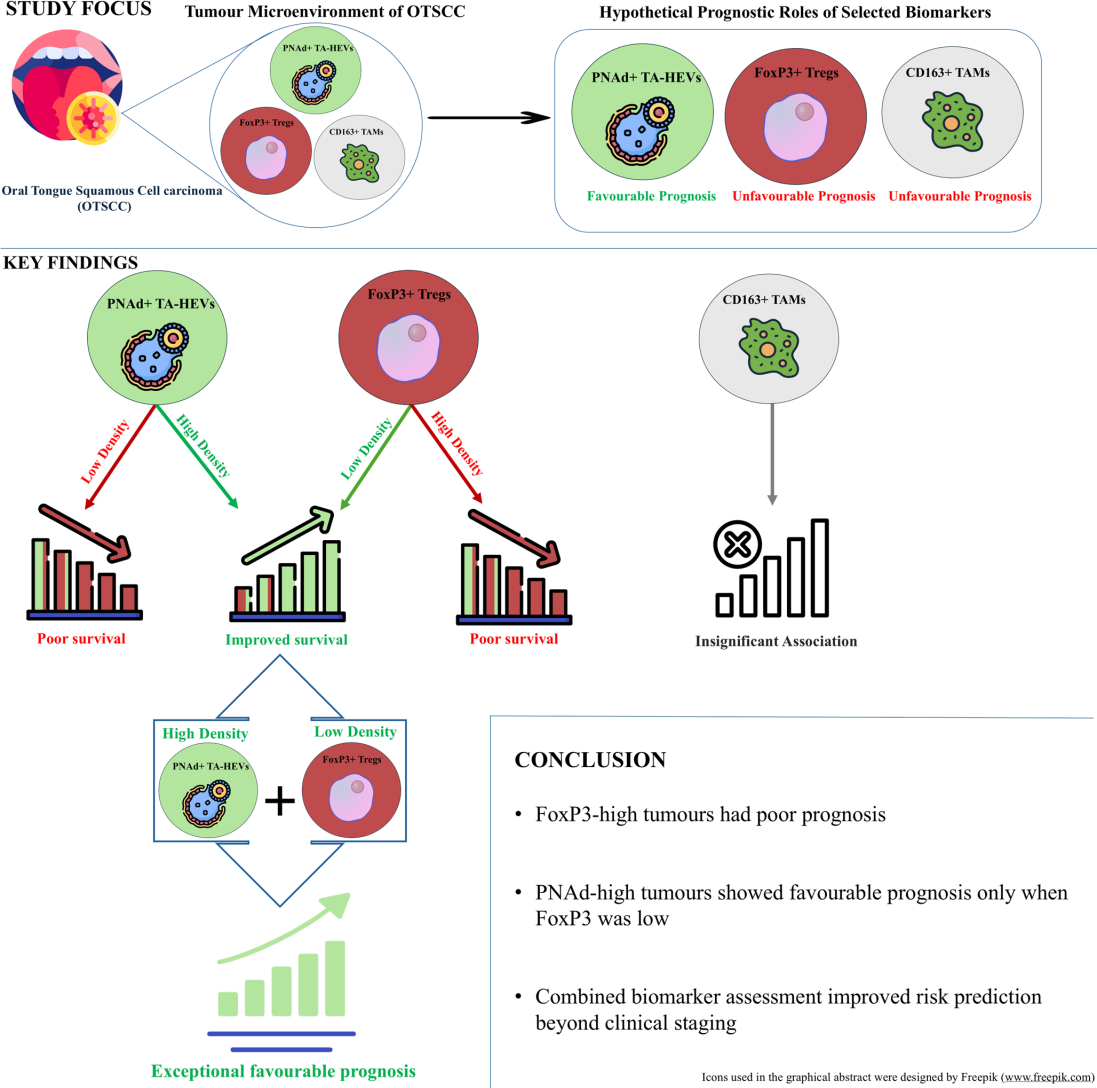

**Supplementary Information:**

The online version contains supplementary material available at 10.1007/s12105-026-01904-4.

## Introduction

Oral squamous cell carcinoma (OSCC) is a major subtype of head and neck squamous cell carcinoma (HNSCC) with the oral tongue (OT) being the most common site, representing approximately 25–40% of cases [1]. These tumours are histologically heterogeneous, locally invasive, and exhibit a high propensity for cervical lymph node (LN) metastasis [2].

Management involves a multidisciplinary approach with surgery as the primary treatment, often combined with radiotherapy. Chemotherapy and immunotherapy are increasingly used, particularly for advanced or recurrent cases [2, 3]. Treatment is largely based on the tumour-node-metastasis (TNM) staging. However, considerable variability in outcomes among patients with identical TNM stages [4, 5] underscores the need for additional prognostic biomarkers that better capture tumour biology and host immune responses [6].

The tumour microenvironment (TME) plays a critical role in OSCC progression and prognosis. Immune markers including tumour-infiltrating lymphocytes, macrophage subsets, Forkhead box Protein 3 (FoxP3) + cells [7, 8], and specialised lymphocyte-recruiting high endothelial venules (HEVs), have been widely investigated using immunohistochemistry (IHC) [5, 9, 10]. Among these, Peripheral Node Addressin (PNAd) + tumour-associated HEVs, which facilitate lymphocyte trafficking, have been associated with favourable outcomes [9–11], whereas CD163+ tumour-associated macrophages (TAMs) have been linked to tumour progression and poor survival in OSCC [12, 13]. FOXP3-expressing cells represent a heterogeneous immune population with variable immunosuppressive activity [14, 15], and controversial prognostic significance. While some HNSCC studies report associations with improved survival [16], OTSCC studies link high FoxP3+ cell density to poorer outcomes [17].

Despite increasing interest in immune biomarkers in OTSCC, critical knowledge gaps persist. Most studies evaluate these markers individually, limiting insight into their biological interplay within the TME [18]. Moreover, disease-specific survival analyses rarely account for competing non-cancer mortality, potentially biasing prognostic estimates in clinically heterogeneous cohorts [19].

This study, therefore, integrates PNAd+ TA-HEVs, CD163+ TAMs, and FoxP3+ cells in a national treatment-naïve OTSCC cohort, evaluating their biological interplay and testing whether a combined assessment improves prognostic stratification beyond conventional staging.

## Methods

### Patient Cohort and Specimens

This work is part of the national, multi-centre, retrospective NORwegian Oral Cancer (NOROC) study [20]. In the present study, we included 126 patients with primary, treatment-naïve SCC of the mobile, anterior two-thirds of the tongue (OTSCC). Patients were diagnosed between January 1st, 2005, and December 31st, 2009, at the Norwegian university hospitals treating OSCC (Bergen, Oslo, Trondheim, and Tromsø). Data were collected from hospital files and pathology reports. The cause of death was retrieved from the Cause of Death Registry if not reported in the patients’ hospital files. The last day of follow-up was June 1st, 2015 [20]. Experienced and calibrated pathologists reclassified the tumours according to the AJCC Cancer Staging Manual, 8th edition [21]. Patient recruitment and data collection methods have been detailed previously [22].

The Regional Committee for Medical and Health Research Ethics of Northern Norway approved the use of patient samples and clinical data (Protocol numbers 2013/1786 and 2015/1381). Patients still alive were given the opportunity to opt out of the study, but the need to obtain written or oral consent was waived. The study broadly follows the Reporting Recommendations for Tumour Marker Prognostic Studies (REMARK) guidelines [23].

### Inclusion and Exclusion Criteria

The inclusion criteria were the availability of tissue from the primary tumours, patient clinical data and follow-up, localisation of SCC on the OT, and treatment with curative intent. Excluded were patients with previous head and neck radiotherapy, previous oral, oropharyngeal or pharyngeal cancer, malignancies with non-SCC histopathology and those receiving palliative treatment or known distant metastasis. Tumours from the base of the tongue were not included, as they are considered a separate entity from OTSCC [24].

### Immunohistochemical Protocols

Immunohistochemistry (IHC) was performed on four micrometre-thick, formalin-fixed, paraffin-embedded (FFPE) tissue sections that were deparaffinised in xylene and rehydrated through graded alcohols prior to staining. Staining was performed on serial sections in independent runs for each biomarker.

### PNAd Immunohistochemical Staining

PNAd staining was performed manually to detect high endothelial venules (HEVs) following established protocols [25]. Tissue sections were subjected to heat-induced epitope retrieval in 0.01 M sodium citrate buffer (pH 6.0). Peroxidase activity was blocked with 3% hydrogen peroxide (H_2_O_2_). Sections were incubated with rat monoclonal anti-PNAd antibody (Clone MECA-79, Cat. No. 120801, BioLegend, San Diego, CA) at 1:25 dilution for 30 min at room temperature. This antibody specifically recognises peripheral node addressin (PNAd), expressed on the luminal surface of HEVs. Detection was performed using HRP-conjugated goat anti-rat immunoglobulin light chain secondary antibody (Cat. No. AP202P, Millipore, Temecula, CA) at 1:250 dilution for 30 min at room temperature. Visualisation was performed using 3,3’-diaminobenzidine (DAB) as the chromogen (Dako EnVision + System, Dako, Glostrup, Denmark). Counterstaining was done with Harris haematoxylin (Sigma-Aldrich, St. Louis, MO). Each staining run included positive control slides (FFPE human lymph node tissue with known HEVs expression) and negative controls (primary antibody omitted) [25].

### FoxP3 Immunohistochemical Staining

FoxP3 staining was performed manually to identify FoxP3+ cells. Tissue sections were subjected to heat-induced epitope retrieval in sodium citrate buffer (pH 6.0). Non-specific protein binding was blocked with 1.5% goat serum before incubation overnight at 4 °C with mouse monoclonal anti-FoxP3 antibody (Clone 236A/E7, Cat. No. ab20034, Abcam, Cambridge, UK) at a 1:50 dilution in 1.5% goat serum/PBS. After rinsing, sections were incubated with HRP-conjugated goat anti-mouse IgG secondary antibody (Cat. No. A-2554, Sigma-Aldrich, St. Louis, MO) at a 1:100 dilution in 1.5% goat serum/PBS for 30 min at room temperature before visualisation with DAB substrate. Sections were counterstained with Harris haematoxylin. Each staining run included positive control slides (human tonsillar tissue with known FoxP3+ cell populations) and negative controls (primary antibody omitted) [26].

### CD163 Immunohistochemical Staining

CD163 staining was performed using the Ventana BenchMark XT automated immunohistochemistry platform (Ventana Medical Systems, Tucson, AZ, USA) at the Diagnostic Clinic-Clinical Pathology, University Hospital of North Norway, as described in established protocols [25]. This IVD-approved system minimises technical variability through automated reagent delivery, precise temperature control, and consistent washing protocols. Tissue sections underwent heat-induced epitope retrieval using CC1 standard pretreatment (Ventana Cell Conditioning Solution, proprietary Tris-based buffer, pH ~ 8.0). A commercially pre-diluted mouse monoclonal anti-CD163 antibody (Clone MRQ-26, Cell Marque/Roche, Cat. No. 760–4437) was applied for 32 min at 37 °C. This antibody is pre-optimised for the Ventana BenchMark platform at a validated concentration. Detection was performed using the Ventana ultraView Universal DAB Detection Kit, which includes HRP-conjugated secondary antibodies. Visualisation employed DAB chromogen. Automated counterstaining was performed using the standard haematoxylin provided with the detection system. Quality control procedures included positive control slides (tonsil or lymph node tissue with known CD163 expression) and negative controls (primary antibody omitted) in each staining run. All CD163-stained sections demonstrated adequate nuclear counterstaining upon direct microscopic evaluation. While published photomicrographs may appear lighter than direct microscopic visualisation due to the modified haematoxylin formulation used in automated systems, microscopic assessment confirmed a staining quality suitable for quantification.

### Quantification Strategy and Image Analysis

Different quantification methods were employed for each biomarker because their morphology and staining patterns were distinct, making different scoring approaches beneficial. All scoring was performed blinded to patient clinical outcomes. Hotspots (n = 5 per marker) were identified independently for each biomarker on serial sections as regions with highest marker-specific density. This approach reflects the distinct spatial distributions of these biomarkers within the TME.

### PNAd+ Tumour Associated High Endothelial Venules (Manual Assessment)

Many HEVs exhibited incomplete vessel wall staining requiring morphological interpretation, precluding reliable automated detection. Therefore, PNAd⁺ tumour-associated HEVs were manually assessed by two calibrated observers (IAA and EHO), consistent with previous HEV studies in OSCC [9, 10, 25]. TA-HEVs were defined as brown PNAd-positive venules with vessel-like morphology located within tumour areas. Hotspots containing the highest TA-HEV density were identified at low magnification (5 ×), and venules were manually counted in five fields per section at 20 × magnification. Discrepant scores were jointly reviewed to reach consensus. Inter-observer agreement was excellent (ICC = 0.997).

### FoxP3+ Cells (Automated Nuclear Segmentation)

FoxP3 exhibits predominantly nuclear localisation, enabling reliable automated cell segmentation. Whole-slide images were analysed using QuPath v0.3.2 [27]. Following haematoxylin/DAB channel separation, positive cell detection parameters were optimised and validated against manual counts in representative regions before establishing a detection threshold of 0.100, with minor adjustments applied in images with strong staining intensity or background signal. Five hotspot annotations (20 × field of view) were placed in regions with the highest FoxP3⁺ cell density within tumour–stromal areas while excluding artefacts and regions of non-specific staining. The mean number of FoxP3⁺ cells across hotspots was calculated for each section.

### CD163+ Tumour-Associated Macrophages (Area Fraction Analysis)

CD163⁺ tumour-associated macrophages frequently exhibited irregular, dendritic morphologies with overlapping cytoplasmic processes, precluding reliable individual cell counting in dense infiltrates. Therefore, CD163 staining was quantified as the area fraction of positive staining using ImageJ/Fiji (v2.9.0), consistent with previously described area-based IHC quantification approaches. Five CD163-rich hotspots per tumour section were analysed at 20 × magnification and converted to 8-bit images. Intensity thresholds were optimised to segment positive staining from background while preserving the analysed tissue area, with minor adjustments applied according to staining intensity following observer consensus. The percentage of thresholded positive area was recorded for each field and averaged per tumour [28].

CD163 quantification was performed as an overall stromal density measure without spatial compartmentalisation. Although the prognostic relevance of CD163⁺ TAMs may vary across tumour compartments (e.g., tumour centre versus invasive front), overall density-based assessment has been widely applied in OSCC studies [5, 13, 29] and was adopted here to maintain methodological consistency with existing literature.

The distribution of hotspots differed for each marker, reflecting their distinct spatial patterns within the TME. To investigate the spatial relationship between PNAd+ TA-HEVs and FoxP3+ cells, images of adjacent sections stained for each marker from a representative OTSCC tissue sample were juxtaposed for visual assessment.

### Cut-off Determination and Sensitivity Analysis

No universally accepted cut-off values exist for PNAd, FoxP3 or CD163 in OTSCC. Published studies employ heterogeneous thresholds (e.g., median splits, ROC-derived values, or arbitrary categories), limiting cross-study comparability [6]. To ensure transparency and reproducibility, we used quintile-based categorisation with the first quintile (20th percentile) as the primary threshold. Tumours were classified as “low” (PNAd ≤ 2.20 HEVs/hotspot, FoxP3 ≤ 72.8 cells/hotspot, CD163 ≤ 6.34% positive area) versus “high” (all higher values), identifying tumours with the lowest biomarker density while retaining adequate sample sizes for multivariable modelling, consistent with REMARK recommendations [23]. Composite variables combining high/low expression of marker pairs were generated to assess synergistic prognostic effects.

To determine whether the prognostic associations were robust, rather than dependent on data-driven thresholds, sensitivity analyses were performed using alternative cut-points and continuous-variable models (Supplementary Methods) [30].

### Clinical and Pathological Variable Coding

Beyond biomarker categorisation, other clinical and pathological variables were coded as follows for multivariable modelling. The primary outcome was five-year disease-specific death (DSD) from OTSCC. Independent variables were the IHC scores (high versus low) for PNAd, CD163 and FoxP3. Covariates were age, sex, T status, N status, and tumour grade. Patients were categorised as younger (< 65 years) or older (≥ 65 years) based on the median age at diagnosis and established geriatric oncology cutoff [31]. T status was used both in its original form and dichotomised into T1 vs T2/T3. N status was based on histopathology (pN) for patients who underwent neck surgery; otherwise, on clinical/radiological examination (cN). It was dichotomised into N0 (no signs of lymph node involvement) and N + (lymph node metastasis). Tumour grade based on histopathological differentiation was categorised as low-grade (moderate and well-differentiated) or high-grade (poorly differentiated) [32].

Composite pathological tumour-node-metastasis (pTNM) stage was defined according to the AJCC 8th Edition. Because very few patients had advanced-stage disease, AJCC Stage III and Stage IV were merged based on predefined criteria into a single “Stage III” category to ensure statistical stability; thus, the three categories used in the models were Stage I, Stage II, and Stage III (III/IV combined). In the primary multivariable models, individual T- and N-status were used as covariates to maximise prognostic granularity and avoid the multicollinearity inherent in composite staging. For nested model comparisons evaluating the incremental prognostic value of immune biomarkers beyond established clinical staging, this composite pTNM variable (Stage I–III, with III/IV combined) was used as the baseline predictor, to represent the established standard for clinical risk stratification [21].

### Statistical Analysis

The intraclass correlation coefficient (ICC) was used to assess the interrater reliability of PNAd+ TA-HEV counting between the two observers, using a two-way mixed-effects model for mean rating (K = 2) and absolute agreement [33]. Variable distributions across IHC scores for PNAd, CD163 and FoxP3 were analysed using cross-tabulations, with Pearson’s chi-square test applied for significance.

The primary event of interest was DSD. The age distribution of this cohort imposes a substantial risk of non-cancer mortality; thus, standard Cox proportional hazards regression would be methodologically inappropriate. Cox models assume non-informative censoring and treat competing deaths (e.g., cardiovascular diseases, other malignancies, comorbid diseases) as if patients remain indefinitely at risk for the event of interest. This violates the independence assumption and leads to systematic overestimation of disease-specific mortality risk, producing biased hazard ratios and cumulative incidence estimates [19, 34].

To address this, we employed competing-risk regression using the Fine-Gray subdistribution hazard model, which explicitly accounts for the mutually exclusive nature of disease-specific versus the non-disease-specific deaths. This approach models the cumulative incidence function (CIF), providing unbiased estimates of OTSCC-specific death while acknowledging that patients who die from competing causes can no longer experience the event of interest. Cumulative incidence functions are the methodologically correct visualisation for competing-risk data [19, 34].

Cumulative incidence curves were generated and compared using univariate Fine-Gray subdistribution hazard model. For multivariable analyses, competing risk regression using Fine-Gray models estimated subdistribution hazard ratios (sHRs) with 95% confidence intervals (CI). The proportional subdistribution hazards assumption was tested using the time-varying covariate (TVC) option. Sensitivity analyses and nested Fine–Gray regression were performed only for biomarkers demonstrating statistically significant associations with disease-specific death in the primary univariate or multivariable competing-risk analyses.

### Incremental Prognostic Value of Biomarkers Beyond pTNM Stage

To determine whether immune biomarkers provided prognostic information beyond established pTNM stage, we compared nested Fine–Gray competing-risk regression models restricted to patients with complete covariate data (n = 112; 34 DSD, 14 competing events). Baseline characteristics did not differ between complete cases and patients with missing FoxP3 data (n = 8; all *p* > 0.05).

The baseline model (Model 1) included age, sex, histological grade, and composite pTNM stage (Stage I, II, III/IV combined). Sequential models added PNAd alone (Model 2), FoxP3 alone (Model 3), or both biomarkers (Model 4). Incremental prognostic value was evaluated using three complementary metrics: Wald tests (independent association), corrected Akaike Information Criterion [AICc; model fit], and Harrell’s concordance index [C-index; discrimination capacity]. Detailed methodology for C-index estimation in competing-risk settings is provided in Supplementary Methods.

Statistical significance was set at *p* < 0.05. Analyses were performed using Stata 18.0 (Stata Corp, College Station, TX, USA) and Microsoft Excel 2411 (Microsoft, Redmond, WA, USA).

## Results

### Patient Characteristics

This study included 126 patients (60% men) diagnosed with OTSCC in Norway from January 1st, 2005, to December 31st, 2009. The mean age at diagnosis was 63.5 years (range 24–90 years). The characteristics of the cohort are summarised in Table 1. At diagnosis, one-third had T1 tumours and 85% had no LN metastasis. Only patients treated with curative intent were included, excluding those with distant metastases. During the five-year follow-up, 30% experienced DSD, while 15 patients (11.9%) died from competing causes.

**Table 1:** OTSCC patients’ characteristics and their association with PNAd, FoxP3, and CD163

	All	PNAd Status			CD163 Status			FoxP3 Status		
	n	Low n (%)	High n (%)	P-value	Low n (%)	High n (%)	P-value	Low n (%)	High n (%)	P-value
All		25 (19.8)	101(80.2)		24 (19.7)	98 (80.3)		23 (19.5)	95 (80.5)	
*Sex*										
Male	76	15 (60.0)	61 (60.4)	.971	12 (50.0)	62 (63.3)	.233	12 (52.2)	60 (63.2)	.332
Female	50	10 (40.0)	40 (39.6)		12 (50.0)	36 (36.7)		11 (47.8)	35 (36.8)	
*Age years*										
< 65	62	11 (44.0)	51 (50.5)	.561	12 (50.0)	48 (49.0)	.788	12 (52.2)	47 (49.5)	.816
≥ 65	64	14 (56.0)	50 (49.5)		12 (50.0)	50 (51.0)		11 (47.8)	48 (50.5)	
*T-status*										
T1	41	7 (28.0)	34 (33.7)	.261	10 (41.7)	28 (28.6)	.329	8 (34.8)	31 (32.6)	.169
T2	53	11 (44.0)	42 (41.6)		7 (29.2)	45 (45.9)		14 (60.9)	35 (36.8)	
T3	29	5 (20.0)	24 (23.8)		6 (25.0)	23 (23.5)		1 (4.4)	26 (27.4)	
Missing	3	2 (8.0)	1 (0.9)		1 (4.2)	2 (2.0)		0	3 (3.2)	
*N-status*										
N0	89	15 (60.0)	74 (73.3)	.372	20 (83.3)	66 (67.4)	.472	17 (73.9)	67 (70.5)	.908
N +	36	10 (40.0)	26 (25.7)		4 (16.7)	31 (31.6)		6 (26.1)	27 (28.4)	
Missing	1	0	1 (1.0)		0	1 (1.0)		0	1 (1.1)	
*Grade*										
Low	111	21 (84.0)	90 (89.1)	.653	22 (91.7)	85 (86.7)	.852	19 (82.6)	84 (88.4)	.665
High	10	3 (12.0)	7 (6.9)		2 (8.3)	8 (8.2)		2 (8.7)	8 (8.4)	
Missing	5	1 (4.0)	4 (3.7)		0	5 (5.1)		2 (8.7)	3 (3.2)	
*FoxP3*										
Low	23	7 (28.0)	16 (15.8)	.240						
High	95	16 (64.0)	79 (78.2)							
Missing	8	2 (8)	6 (6.0)							
*CD163*										
Low	24	6 (24.0)	18 (17.8)	.158				7 (30.4)	15 (15.8)	.009^*^
High	98	17 (68.0)	81 (80.2)					15 (65.2)	79 (83.2)	
Missing	4	2 (8.0)	2 (2.0)					1 (4.4)	1 (1.0)	

N0, no lymph node metastases. N + : Lymph node metastases. N: number of patients. PNAd (n = 126), FoxP3 (n = 118) and CD163 (n = 122). * *p* < 0.05; ** *p* < 0.01; *** *p* < 0.001

### Identification, Distribution and Staining Characteristics of PNAd+ TA-HEVs, CD163+ TAMs, and FoxP3+ Cells in OTSCC Tissues

**Fig. 1:**
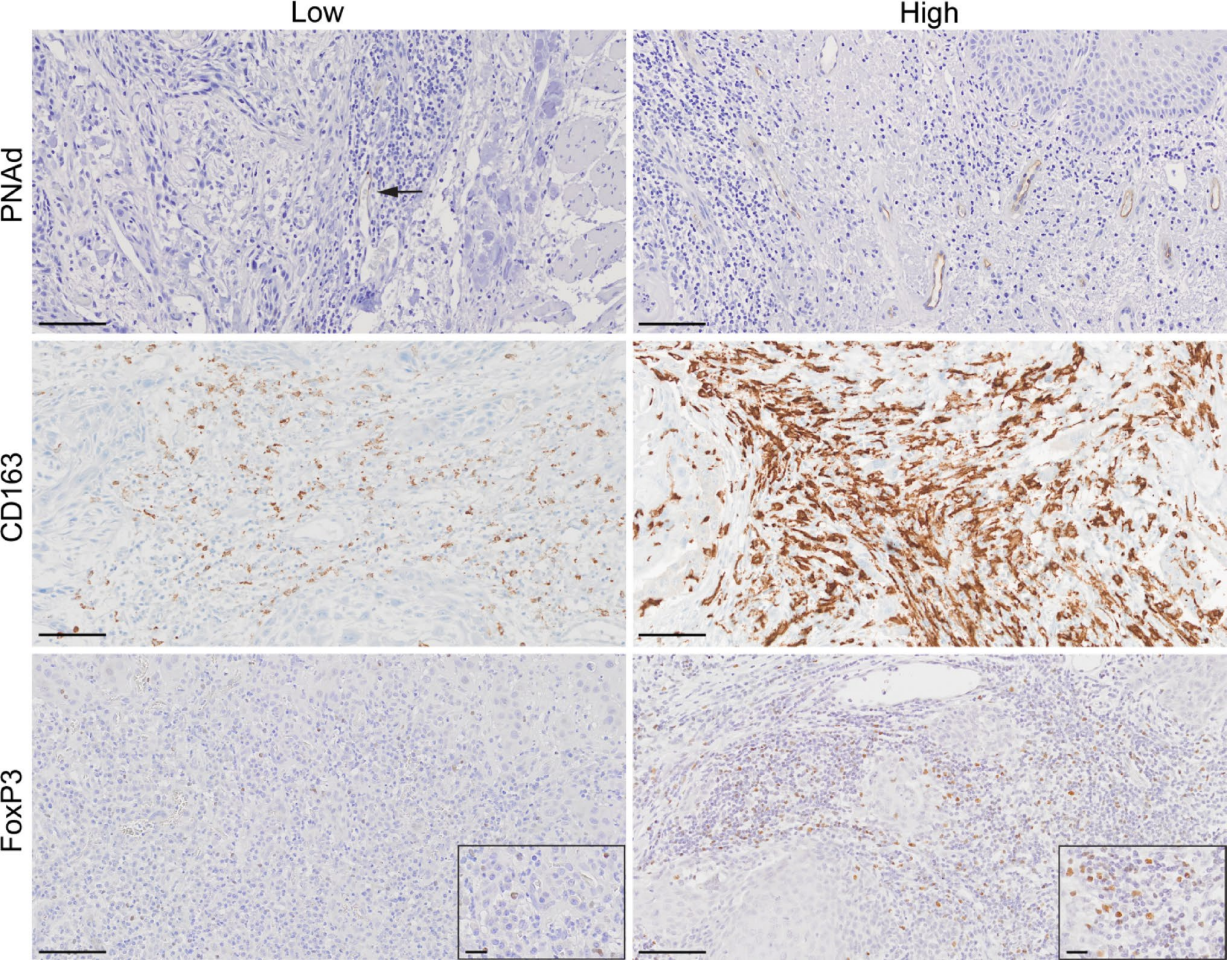
Representative images of OTSCC tissue sections immunohistochemically stained for PNAd (n = 126), CD163 (n = 122), and FoxP3 (n = 118). Each biomarker is represented by images of both low and high score cases. The scale bar indicates 100 µm

PNAd+ TA-HEVs were easily identifiable as brown-stained vessel structures with or without a visible lumen (Fig. 1). The vessel wall thicknesses varied, and staining was sometimes patchy. Most PNAd+ TA-HEVs were located in areas with dense lymphocyte infiltration, with only one specimen negative for PNAd. Areas rich in PNAd+ TA-HEVs were predominantly found at the tumour periphery, and towards the tumour surface. The intraclass correlation coefficient of the two observers’ PNAd score was 0.997 (95% CI: 0.995–0.998) [33]. In most tumours, CD163 staining was prominent, with positive cells densely distributed throughout the tumour stroma, including the periphery, invasive front, and stroma surrounding tumour islands. CD163 staining was observed in both the cell membrane and cytoplasm of irregularly shaped cells (Fig. 1). FoxP3+ cells were observed in lymphocyte-rich stromal areas, including the tumour periphery, invasive front and stroma around tumour islands. FoxP3+ cells were typically small with dark and round nuclei, in accordance with the morphology of lymphocytes (Fig. 1). Negative controls displayed no staining, whereas positive controls showed staining as expected for the various antibodies.

Visual inspection of serial adjacent sections stained for FoxP3 and PNAd revealed the distribution of FoxP3+ cells and PNAd+ TA-HEVs within the same TME niche (Supplementary Figure S1). This qualitative observation complements our broader findings, though formal co-localisation studies would be needed to definitively establish spatial relationships.

### Associations with Clinicopathological Variables

Table 1 summarises the characteristics of OTSCC patients and their associations with PNAd, CD163, and FoxP3. None of the markers showed significant associations with age, sex, T status, N status, or histologic grade (all *p* > 0.05). FoxP3 was significantly associated with CD163 (*p* = 0.009), with high FoxP3 more frequent in tumours with high CD163 expression, suggesting a co-occurrence of immunosuppressive FoxP3+ cells and CD163+ TAMs. In contrast, PNAd showed no significant association with FoxP3 (*p* = 0.240) or CD163 (*p* = 0.158).

### Biomarker Distribution by Patient Outcome

Patient characteristics stratified by outcome are presented in Table 2. Older age (*p* = 0.007), nodal involvement (*p* = 0.013), and high tumour grade (*p* = 0.028) were significantly associated with disease-specific mortality, while T-status showed a marginal association (*p* = 0.052). For immune biomarkers, PNAd and FoxP3 expression were significantly associated with outcomes (*p* = 0.027 and *p* = 0.025, respectively), whereas CD163 showed no significant association (*p* = 0.517). Notably, high PNAd expression was observed in all patients who died from competing causes (100%), while low PNAd expression was more frequent among OTSCC-specific deaths (31.6% vs. 17.8% in survivors). Similarly, high FoxP3 expression was more prevalent in OTSCC deaths (92.1%) compared to survivors (65.7%).

**Table 2:** OTSCC patients’ characteristics by outcome

Variable	All n (%)	Alive n (%)	OTSCC death n (%)	Other death n (%)	P-value
*Sex*					
Male	76 (60.3)	45 (61.6)	24 (63.2)	7 (46.7)	0.509
Female	50 (39.7)	28 (38.4)	14 (36.8)	8 (53.3)	
*Age*					
< 65 years	62 (49.2)	42 (57.5)	18 (47.4)	2 (13.3)	0.007^*^
≥ 65 years	64 (50.8)	31 (42.5)	20 (52.6)	13 (86.7)	
*T-status*					
T1	41 (32.5)	29 (39.7)	6 (15.8)	6 (40.0)	
T2	53 (42.1)	28 (38.4)	19 (50.0)	6 (40.0)	0.052
T3	29 (23.0)	16 (21.9)	10 (26.3)	3 (20.0)	
Missing	3 (2.4)	0	3 (7.9)	0	
*N-status*					
N0	41 (32.5)	29 (39.7)	6 (15.8)	6 (40.0)	
N +	82 (65.1)	44 (60.3)	29 (76.3)	9 (60.0)	0.013^*^
Missing	3 (2.4)	0	3 (7.9)	0	
*Grade*					
Low	111 (88.1)	69 (94.0)	29 (76.3)	13 (86.6)	
High	10 (7.9)	2 (3.0)	7 (18.4)	1 (6.7)	0.028^*^
Missing	5 (4.0)	2 (3.0)	2 (5.3)	1 (6.7)	
PNAd, m (SD)	3.8 (1.8)	3.9 (1.8)	3.4 (2)	4.2 (1.2)	0.180
*PNAd*					
Low	25 (19.8)	13 (17.8)	12 (31.6)	0	0.027^*^
High	101 (80.2)	60 (82.2)	26 (68.4)	15 (100)	
CD163, m (SD)	10.5 (5.4)	10.4 (5.8)	10.9 (5.3)	9.6 (4.1)	0.743
*CD163*					
Low	24 (19.1)	14 (19.2)	5 (13.2)	5 (33.3)	
High	98 (77.7)	56 (76.7)	32 (84.2)	10 (66.7)	0.517
Missing	4 (3.2)	3 (4.1)	1 (2.6)	0	
FoxP3, m (SD)	208 (158.2)	192.3 (143.4)	228.8 (172.4)	226.2 (186)	0.478
*FoxP3*					
Low	23 (18.2)	18 (24.7)	2 (5.3)	3 (20.0)	
High	95 (75.4)	48 (65.7)	35 (92.1)	12 (80.0)	0.025^*^
Missing	8 (6.4)	7 (9.6)	1 (2.6)	0	

N0, no lymph node metastases; N + , Lymph node metastases; N, number of patients; Alive, patients alive throughout the study period; OTSCC death, patients who died from OTSCC; Other death, patients who died from other causes (competing event). * *p* < 0.05; ** *p* < 0.01; *** *p* < 0.001

### Competing Risk Survival Analyses

#### Univariate Analyses

Univariate Fine–Gray subdistribution hazard analyses identified several clinicopathological and immune variables associated with five-year DSD (Table 3). Advanced tumour stage (T2 and T3 vs. T1), nodal metastasis (N + vs. N0), and high tumour grade were all significantly associated with poorer outcomes (all *p* < 0.05). Among immune markers, high FoxP3+ cell density emerged as the strongest prognostic factor (sHR 5.11, 95% CI 1.26–20.73, *p* = 0.022), whereas low PNAd + TA-HEV density was also associated with increased risk (sHR 2.25, 95% CI 1.13–4.46, *p* = 0.020). Age, sex, and CD163+ TAM density were not significantly associated with DSD.

**Table 3:** Unadjusted 5-year DSD risk in OTSCC patients

	Unadjusted sHR (95% CI)	P-value
*All*		
*Sex*		
Male	1.12 (0.58, 2.16)	.738
Female	Reference	
*Age*		
< 65 years	Reference	.763
≥ 65 years	1.10 (0.56, 2.07)	
*T-status*		
T1	Reference	
T2	2.82 (1.15, 6.89)	.023^*^
T3	2.87 (1.04, 7.88)	.041^*^
*N-status*		
N0	Reference	< .001^***^
N +	3.67 (1.95, 6.92)	
*Grade*		
Low	Reference	.001^**^
High	3.88 (1.72, 8.73)	
*PNAd*		
Low	2.25 (1.13, 4.46)	.020^*^
High	Reference	
*FoxP3*		
Low	Reference	.022^*^
High	5.11 (1.26, 20.73)	
*CD163*		
Low	Reference	.260
High	1.71 (0.67, 4.33)	

The subdistribution hazard ratio (sHR) of DSD with 95% CI as well as the statistical significance of differences across variable categories were calculated using univariate Gray-fine subdistribution hazard model. sHR: subdistribution hazard ratio. N0: no lymph node metastases. N + : Lymph node metastases. * *p* < 0.05; ** *p* < 0.01; *** *p* < 0.001

**Fig. 2:**
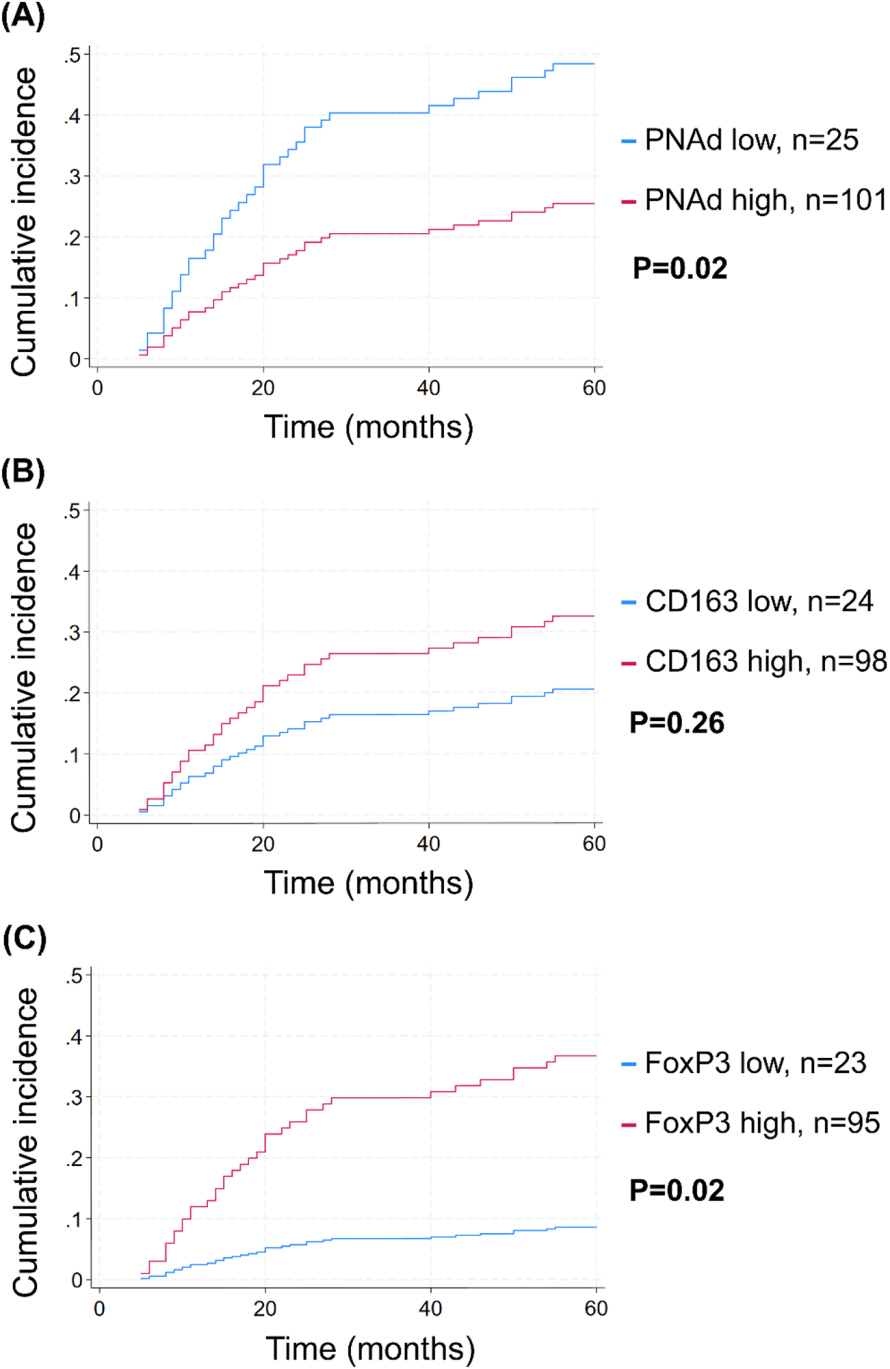

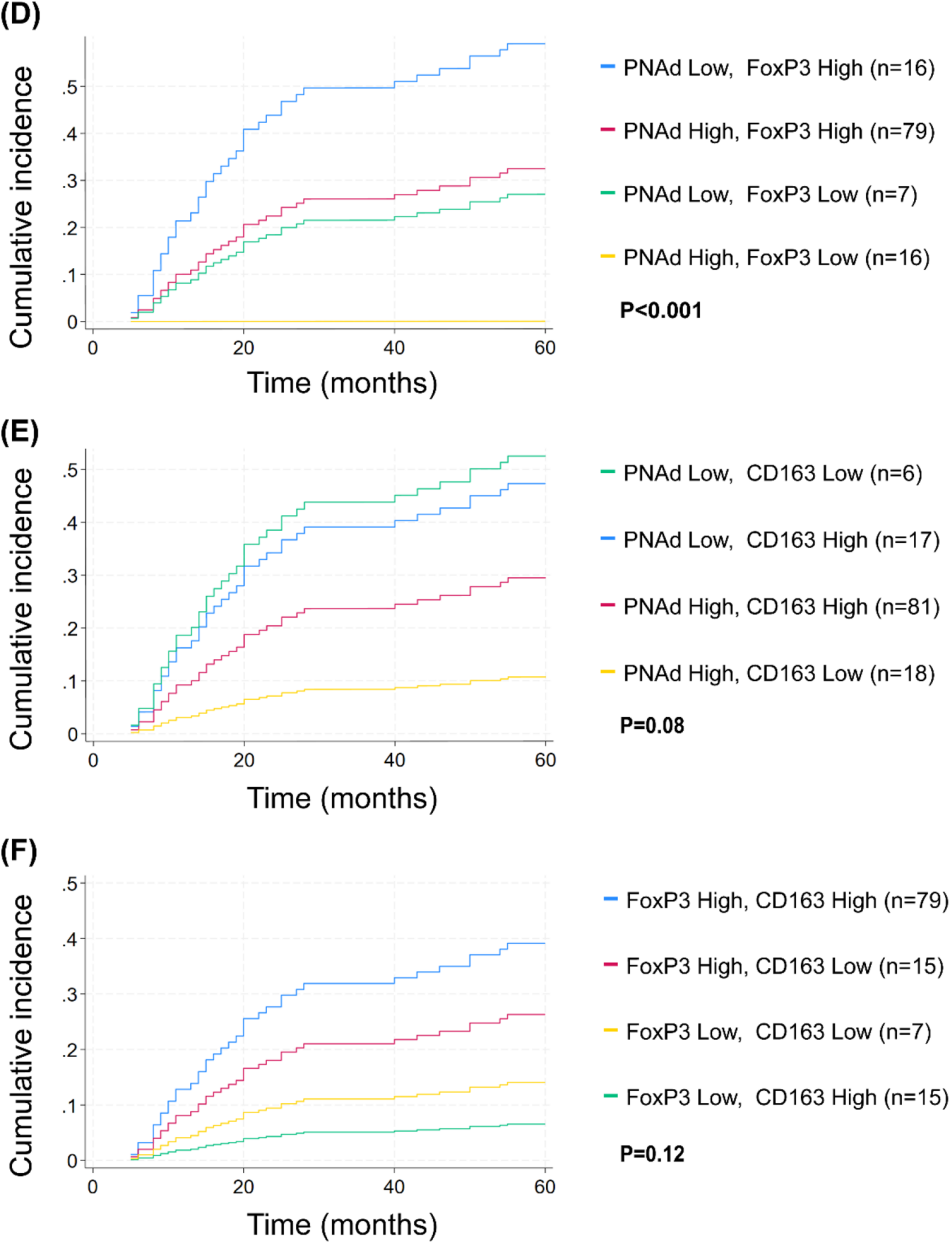
Univariate cumulative incidence curves for five-year disease-specific death in OTSCC patients stratified by biomarker expression. **A–C** Individual markers: **A** TA-HEVs (N = 126), **B** CD163 (N = 122), (**C**) FoxP3 (N = 118). **D–F** Combined marker profiles: **D** TA-HEVs and FOXP3 (N = 118), **E** TA-HEVs and CD163 (N = 122), and **F** FoxP3 and CD163 (N = 116). *P*-values were estimated using a univariate comparison of curves

Figure 2 displays cumulative incidence curves for five-year DSD based on biomarker scores: A) PNAd, which was significantly associated with lower DSD risk (*p* = 0.02), B) CD163, which showed no significant association (*p* = 0.26) and C) FoxP3, which was significantly associated with higher DSD risk (*p* = 0.02).

#### Combined Biomarker Profiles

To assess whether combinations of biomarker scores could enhance prognostic stratification, we evaluated cumulative incidence curves for all pairwise marker combinations (Fig. 2D, E, F). The combination of high PNAd and low FoxP3 was associated with particularly favourable outcomes, with no DSD observed in this group, whereas patients with low PNAd and high FoxP3 experienced the highest DSD rates (Fig. 2D). In contrast, combinations involving CD163 (PNAd-CD163, Fig. 2E; FoxP3-CD163, Fig. 2F) showed lower prognostic discrimination than PNAd and FoxP3 assessed individually (Fig. 2C), suggesting that CD163 does not enhance the prognostic value of the other markers.

#### Multivariable Analyses

Multivariable Fine-Gray subdistribution hazard models were constructed for individual biomarkers as well as for a combined model including PNAd and FoxP3, as these biomarkers seemed to enhance each other’s prognostic potential (Table 4). No violations of the proportional hazard assumption were detected.

**Table 4:** Multivariable competing risk analyses

	Adjusted^1^ sHR (95% CI)	Adjusted^2^ sHR (95% CI)	Adjusted^3^ sHR (95% CI)	Adjusted^4^ sHR (95% CI)
*Sex*				
Male	1.01 (0.46, 2.21)	1.06 (0.49, 2.30)	1.05 (0.49, 2.23)	1.00 (0.48, 2.09)
Female	Reference	Reference	Reference	Reference
*Age*				
< 65 years	Reference	Reference	Reference	Reference
≥ 65 years	1.35 (0.62, 2.92)	1.10 (0.48, 2.49)	1.25 (0.55, 2.84)	1.03 (0.46, 2.32)
*T-status*				
T1	Reference	Reference	Reference	Reference
T2/T3	2.06 (0.73, 5.83)	2.34 (0.89, 6.15)	1.97 (0.72, 5.36)	2.19 (0.81, 5.97)
*N-status*				
N0	Reference	Reference	Reference	Reference
N +	2.85^*^ (1.18, 6.92)	2.95^*^ (1.24, 7.00)	2.51 (0.96, 6.56)	3.21^*^ (1.36, 7.60)
*Grade*				
Low	Reference	Reference	Reference	Reference
High	2.86^*^ (1.04, 7.86)	3.33^*^ (1.05, 10.55)	3.32^*^ (1.13, 9.78)	3.61^*^ (1.22, 10.68)
*PNAd*				
Low	1.52 (0.69, 3.33)			2.58^*^ (1.25, 5.34)
High	Reference			Reference
*FoxP3*				
Low		Reference		Reference
High		5.40^*^ (1.92, 15.17)		8.78^*^ (3.53, 21.87)
*CD163*				
Low			Reference	
High			1.58 (0.62, 4.02)	

The subdistribution hazard ratio (sHR) of 5-year DSD with 95% CI was calculated using Fine-Gray subdistribution hazard models. sHR: subdistribution hazard ratio. Adjusted^1^: separate model for PNAd; Adjusted^2^: separate model for FoxP3; Adjusted^3^: separate model for CD163; Adjusted^4^: combined model including PNAd and FoxP3, excluding CD163. * Statistically significant (95% CI does not cross 1.0; *p* < 0.05)

FoxP3+ cell density was significantly associated with DSD when analysed alone (sHR = 5.40, 95% CI: 1.92–15.17) and in combination with PNAd (sHR = 8.78, 95% CI: 3.53–21.87). CD163 showed no significant association with DSD in either univariate or multivariable models and was therefore excluded from subsequent nested Fine–Gray regression and sensitivity analyses, which focused on PNAd⁺ TA-HEVs and FoxP3⁺ cells. PNAd demonstrated prognostic significance only when included alongside FoxP3 (sHR = 2.58, 95% CI: 1.25–5.34).

#### Incremental Prognostic Value of Immune Biomarkers

To determine whether these biomarkers added prognostic value beyond established clinicopathological parameters, nested Fine–Gray models were constructed using composite pTNM stage as the baseline. Model performance was evaluated using AICc, Wald tests, and the C-index. As presented in Table 5, the baseline clinical model (Model 1) achieved moderate discrimination (C-index = 0.727, AICc = 300.46). Adding PNAd alone produced minimal improvement (Model 2: C-index = 0.736, ΔAICc =  − 0.10, *p* = 0.104), whereas adding FoxP3 alone substantially improved model fit and discrimination (Model 3: C-index = 0.770, ΔAICc =  − 5.23, *p* = 0.003). The combined biomarker model achieved the highest performance (Model 4: C-index = 0.784, ΔAICc =  − 8.93, joint Wald χ^2^ = 25.90, *p* < 0.0001). The C-index improvement (Δ =  + 0.057) exceeded the clinically meaningful threshold (≥ 0.05), with FoxP3 providing consistent independent prognostic value and PNAd showing context-dependent significance in the presence of FoxP3. Detailed model coefficients are provided in Supplementary Table S2.

**Table 5:** Nested Fine–Gray competing-risk models: incremental prognostic value of immune biomarkers

Model	Variables included	AICc	Δ AICc (vs M1)	Wald χ^2^ (*p*-value)	C-index (discrimination)
Model 1 (Baseline)	Age, Sex, Grade, pTNM Stage	300.46	Ref	—	0.727
Model 2	Baseline + PNAd	300.36	− 0.10	2.65 (*p* = 0.104)	0.736 (Δ = + 0.009)
Model 3	Baseline + FoxP3	295.23	− 5.23	8.81** (*p* = 0.003)	0.770 (Δ = + 0.043)
Model 4 (Combined)	Baseline + PNAd + FoxP3	291.53	− 8.93	25.90*** (*p* < 0.0001)	0.784 (Δ = + 0.057)

Fine–Gray subdistribution hazard regression analysis for disease-specific death (Event Type 1) with non-disease-related death as a competing risk (Event Type 2). Models were adjusted for age, sex, tumour grade, and pTNM stage (I, II, III/IV merged). Analyses included patients with complete covariate data (n = 112; 34 disease-specific deaths, 14 competing events). ΔAICc indicates change relative to Model 1. Wald χ^2^ evaluates the incremental contribution of biomarkers beyond clinical variables. C-index represents model discrimination estimated from cause-specific Cox regression (see Methods and Supplementary Information). **p < 0.01; ***p < 0.001

#### Sensitivity Analyses for Cut-off Robustness

Sensitivity analyses using alternative cut-points and continuous-variable models confirmed the robustness of these findings (Supplementary Table S1). FoxP3 retained prognostic significance across all models, while PNAd demonstrated context-dependent effects consistent with biological synergy with FoxP3.

## Discussion

In this study, we assessed the prognostic value of TA-HEVs, CD163+ TAMs and FoxP3+ cells using IHC staining for PNAd, CD163, and FoxP3, respectively. A high number of FoxP3+ cells in the TME was an independent predictor of increased DSD risk within five years of OTSCC diagnosis. PNAd demonstrated context-dependent prognostic value, with significant associations in univariate analyses and in multivariable models that included FoxP3, but not when adjusted for clinical covariates alone. CD163+ TAM density showed no significant association with DSD in any analysis.

These findings indicate complex immunological interactions within the OTSCC TME, where a dynamic balance between anti- and pro-inflammatory immune populations shape disease progression and patient outcomes [8]. Transformed cells express neoantigens that evoke an adaptive immune response, which may suppress tumour growth [8, 16]. Cytotoxic T cells are key players in this response, and they can enter the TME through TA-HEVs [35]. However, the antitumour immune response often diminishes over time, partly due to anti-inflammatory cells like TAMs and regulatory T cells (Tregs), which create an immunosuppressive TME [36]. FoxP3, a transcription factor that serves as a master regulator of Treg development and function, characterises these immunosuppressive lymphocytes [37]. Furthermore CD163+ TAMs release cytokines such as IL-10 and TGF-β, crucial for recruiting and activating Tregs which inhibit effector T cell proliferation in solid tumours [38]. Moreover, emerging evidence indicates that this relationship is bidirectional. For instance, *Sun *et al., demonstrated that activated Tregs can actively promote TAM polarisation via Treg-derived cytokines, establishing a positive-feedback loop that further enhances immunosuppression [39]. This mutual reinforcement likely contributes to the evolving immunosuppressive milieu in OTSCC.

Our study identified a trend of higher FoxP3+ cell density in T3 tumours than in T1 or T2 tumours, suggesting a shift towards a more immunosuppressive TME as tumours progress, consistent with Tregs’ role in immunosuppression [40]. However, no significant associations were found between any of the biomarkers (PNAd, CD163 or FoxP3) and clinicopathological parameters (T stage, N stage or tumour grade). Furthermore, PNAd scores showed no significant association with FoxP3+ cell or CD163+ TAM densities, indicating no detectable relationship between PNAd expression and FoxP3+ or CD163+ immune cell infiltration within the TME.

A significant association was observed between FoxP3 and CD163 scores (p = 0.009), suggesting a mutual interplay that contributes to an immunosuppressive environment and tumour progression [39, 41]. This aligns with established models demonstrating that Tregs and TAMs form a bidirectional, reinforcing network across various cancers [38, 39]. These findings highlight the complex immune interactions in OTSCC, where FoxP3+ cells seem to play a significant role in larger tumours, whereas PNAd + TA-HEVs and CD163+ TAMs may have more subtle or independent roles.

The prognostic significance of FoxP3+ cells observed in our cohort aligns with most oral tongue-specific literature but requires contextual nuance. Although Treg infiltration shows variable associations across cancer types [16], a meta-analysis of seventeen solid cancers reported high Treg density to be associated with poor prognosis [40]. Consistent with our findings, most OTSCC studies report that low FoxP3+ cell levels are associated with longer survival [17]. However, broader reviews indicate that the prognostic impact of Tregs is highly context-dependent across cancer types [42]. Importantly, although FoxP3 immunostaining does not exclusively identify functional Tregs, studies in broader head and neck cancer cohorts have reported associations between tumour-infiltrating FoxP3⁺ cells and favourable outcomes [40].

In contrast to the robust independent prognostic value of FoxP3, PNAd demonstrated a more nuanced, context-dependent association, with its prognostic contribution emerging only when evaluated alongside FoxP3. This synergy between FoxP3+ cells and PNAd + TA-HEVs underscores the importance of integrated immune microenvironment profiling over single-marker assessment for robust prognostic evaluation in OTSCC. This concept aligns with mounting evidence that complex immune contextures and tertiary lymphoid structures are key determinants of cancer outcomes [43]. Our findings show that FoxP3+ cells and PNAd + TA-HEVs can localise within the same tumour microenvironment niches (Supplementary Figure S1); although double staining was not performed, this spatial proximity supports the hypothesis of functional interaction. Mouse models suggest Tregs may suppress HEV neogenesis [44]; our observation that tumours with low FoxP3 and high PNAd have favourable prognosis is consistent with this proposed mechanism, though causality cannot be established from observational data. Evaluating HEVs and FoxP3+ cells together may therefore have particular prognostic value, potentially identifying tumours transitioning between tumour-suppressive and tumour-promoting immune responses.

To validate the clinical relevance of this biological synergy, we conducted comprehensive nested model comparisons demonstrating that the combined PNAd–FoxP3 profile provides incremental prognostic value beyond pTNM stage and tumour grade. The combined biomarker model showed improvements across three complementary metrics: model fit (ΔAICc =  − 8.93), independent association (joint Wald χ^2^ = 25.90, *p* < 0.0001), and discrimination (C-index improved from 0.727 to 0.784, Δ =  + 0.057). This improvement in C-index exceeds the threshold generally considered clinically meaningful (ΔC ≥ 0.05), demonstrating that integrated immune profiling provides discriminatory value beyond pathological staging alone [45]. The convergence of biological plausibility (mechanistic interactions), statistical significance (multiple metrics), and clinical meaningfulness (discrimination improvement) provides robust evidence that integrated FoxP3-PNAd assessment captures prognostic information not fully reflected by established clinicopathological parameters.

Unlike PNAd and FoxP3, CD163 showed no significant association with DSD in our analyses. CD163 was nevertheless included as one of the most widely investigated TAM biomarkers in head and neck cancer, enabling comparison with prior studies employing overall density-based assessments [13, 46]. Several factors may explain this null finding. First, we quantified overall stromal CD163 density without spatial compartmentalisation. Emerging evidence suggests CD163 prognostic value varies by location, as TAMs macrophages at the invasive front may promote progression differently than those in the tumour centre [12, 13, 47]. Our approach may have missed compartment-specific effects. A second consideration is that CD163 represents only one TAM marker; comprehensive profiling of macrophage subsets (M2a–M2d) using multi-marker panels (e.g., CD206, CD204) might reveal associations not captured by CD163 alone [47]. Third, our sample size (n = 122) may have been underpowered to detect modest effects. These considerations highlight that overall CD163 density may be insufficient for prognostic assessment, though our null results remain informative for the field.

### Clinical and Translational Implications

Management of OTSCC relies primarily on surgery with adjuvant radiotherapy, underscoring the need for biomarkers that improve postoperative risk stratification. This treatment, particularly radiation, is associated with chronic side effects such as xerostomia, dysphagia, and osteoradionecrosis, which can severely impact patients’ quality of life [48]. Therefore, improved identification of patients most likely to derive survival benefit from adjuvant radiation remains clinically important.

Clinical parameters, such as the TNM stage, do not always provide a clear indication for adjuvant radiation, as tumours with the same stage can exhibit varying levels of aggressiveness. Our findings suggest that immune biomarkers could refine risk stratification within pTNM stage groups. For example, Stage II patients with high-risk immune profiles (low PNAd, high FoxP3) might benefit from treatment intensification, while those with favourable immune profiles may represent lower-risk disease despite advanced pathological stages. If validated in larger studies, these biomarkers could aid clinicians in making informed treatment decisions and identifying patients requiring intensive follow-up. Both PNAd and FoxP3 staining were distinct and readily quantifiable, using manual or digital scoring approaches, supporting feasibility within routine pathology workflows.

### Study Strengths and Limitations

This study has several methodological strengths. First, the national, multi-centre population-based design eliminates referral bias and enhances generalisability. Second, competing-risk regression provides unbiased disease-specific estimates in a cohort with substantial non-OTSCC mortality (11.9% of deaths were competing causes), addressing a critical methodological gap in biomarker validation studies that typically use conventional survival analysis. Third, simultaneous evaluation of PNAd + TA-HEVs, CD163+ TAMs, and FoxP3+ cells enables direct assessment of their prognostic interplay within the TME. Fourth, the homogeneous treatment naïve cohort eliminates confounding from heterogeneous systemic therapies. Finally, histopathological re-evaluation and systematic outcome ascertainment through national registry linkage ensure data quality. Sensitivity analyses demonstrated that key findings were robust across multiple analytic approaches (Supplementary Table S1), validating our categorisation strategy while illustrating how cut-off point selection influenced discrimination and power.

The main limitation is the cohort size, which may have led to underpowered analyses for CD163 and wide confidence intervals for some parameters, particularly FoxP3. The events-per-variable ratio in the combined model (34 events ÷ 7 parameters = 4.9) is below the commonly recommended threshold of 10, which may result in overfitted effect sizes [49]. Accordingly, independent validation in adequately powered external cohorts is required before clinical implementation of the proposed biomarker framework.

Furthermore, reliance on single markers (CD163 for TAMs, FoxP3 for Tregs) may not fully capture the complexity and heterogeneity of these immune populations. FoxP3 expression is not exclusive to Tregs [14, 15], limiting specificity. Similarly, CD163 does not capture TAM heterogeneity [47]. While we observed FoxP3+ cells and PNAd + TA-HEVs colocalised within TME niches, the absence of double staining limited direct evidence of their spatial interaction.

## Conclusion

High densities of FoxP3+ cells independently predicted increased five-year DSD in OTSCC, indicating an adverse immunoregulatory TME. PNAd + TA-HEVs demonstrated context-dependent prognostic effects, becoming significant only when evaluated alongside FoxP3 expression, consistent with biologically meaningful immune–vascular interactions. Combined biomarker assessment provided incremental prognostic value beyond established clinicopathological parameters, suggesting that integrated immune profiling captures TME features not reflected by conventional staging and may improve risk stratification in treatment-naïve OTSCC. These findings support further investigation of integrated immune profiling as a framework for refined patient stratification and future personalised management strategies, pending external validation.

## Supplementary Information

Below is the link to the electronic supplementary material.

Supplementary Material 1

Supplementary Material 2

## Data Availability

The datasets generated and analysed during the current study are not publicly available due to General Data Protection Regulation (GDPR) restrictions. However, aggregated and de-identified data may be made available from the corresponding author upon reasonable request.

## Abbreviations

AJCC: American Joint Committee on Cancer
CD163: Cluster of Differentiation 163
CI: Confidence interval
CIF: Cumulative incidence function
cN: Clinical N
DSD: Disease specific death
FFPE: Formalin-fixed, paraffin-embedded
FoxP3: Forkhead box P3 protein
HNSCC: Head and neck squamous cell carcinoma
sHR: Subdistribution hazard ratio
HRP: Horse Radish Peroxidase
ICC: Intraclass Correlation Coefficient
IgG: Immunoglobulin G
IgM: Immunoglobulin M
IHC: Immunohistochemistry
LN: Lymph node
M1: Classically activated Macrophages
M2: Alternatively activated macrophages
MECA 79: Mouse Endothelial Cell Antigen-79
NOROC: Norwegian Oral cancer study
OT: Oral Tongue
OSCC: Oral squamous cell carcinoma
pN: Pathological N
PNAd: Peripheral Lymph Node Addressin
REMARK: Recommendation for Tumour Marker Prognostic Studies
TA-HEVs: Tumour associated-High Endothelial Venules
TAMs: Tumour associated Macrophages
TNM: Tumour, Node, Metastasis
TME: Tumour Microenvironment
Tregs: Regulatory T cells
TVC: Time varying covariates

## Code Availability

Code availability is not applicable.
