# Supplementary material for: FoxP3+ Cells and PNAd+ Tumour-Associated High Endothelial Venules: Synergistic Prognostic Markers in Oral Tongue Squamous Cell Carcinoma
Source: Head Neck Pathol. 2026 Mar 26;20(1):35. doi: 10.1007/s12105-026-01904-4 (PMC13022141; doi:10.1007/s12105-026-01904-4)
Supplement: Supplementary file 1 — Supplementary Material 1 [file 12105_2026_1904_MOESM1_ESM.docx]

**SUPPLEMENTARY METHODS**

**FoxP3+ regulatory T cells and High Endothelial Venules: Synergistic and Divergent Prognostic Markers in Oral Tongue Squamous Cell Carcinoma**

Ibrahim Afolabi Abdulsalam^1*^, Kjersti Sellæg^1^, Faith O. Benebo^2^, Nancy C. Ojei^2^, Sonja E. Steigen^1,3^, Lars Uhlin-Hansen^1,3^, Inger-Heidi Bjerkli^1^, Anna M. Wirsing^1,3^, Synnøve Norvoll Magnussen^1^, Elin Hadler-Olsen^1,4*^

^1^Department of Medical Biology, UiT – The Arctic University of Norway, Tromsø, Norway; ^2^Department of Community Medicine, UiT – The Arctic University of Norway, Tromsø, Norway; ^3^Department of Clinical Pathology, University Hospital of North Norway, Tromsø, Norway, ^4^The Public Dental Health Service Competence Center of Northern Norway, Tromsø

*Corresponding author:

Ibrahim Afolabi Abdulsalam

Email: [ibrahim.abdulsalam@uit.no](mailto:ibrahim.abdulsalam@uit.no)

ORCID iD: 0009-0009-9553-3355

## Methods

### Immunohistochemical staining

CD163 staining was performed with the automated Ventana Benchmark, XT (Ventana, Tucson, AZ, USA) IHC slide staining system at the Diagnostic Clinic-Department of Clinical Pathology, University Hospital of North Norway. A Ventana Enhanced diaminobenzidine (DAB) detection kit (Ventana, Tucson, AZ) and a commercially available prediluted monoclonal mouse primary CD163 antibody (Clone MRQ-26 from Roche, Cell Marque, Cat. No: 760-4437) were used. Staining was done following standard procedures where deparaffinized and blocked sections were heated in 0.01 M sodium citrate buffer at pH 6.0 for antigen retrieval. For visualization, an antibody cocktail of horseradish peroxidase (HRP)-labelled goat anti-mouse IgG/IgM and mouse anti-rabbit secondary antibodies (Ventana UltraView Universal DAB Detection Kit, Roche, Mannheim, Germany; Cat. No. 760–500) were used, along with diaminobenzidine. The duration of secondary antibody incubation was regulated by the manufacturer. Each run included a control slide with known positivity for each antibody (tonsil or lymph node) [1].

For PNAd and FoxP3 staining, tissue sections were incubated at 60°C for at least one hour or overnight, then deparaffinized in xylene and rehydrated in a graded ethanol. Antigen retrieval involved boiling in sodium citrate buffer (pH 6.0) for 20 minutes, and 3% H_2_O_2_ (Dako Glostrup, Denmark) was used to block endogenous peroxidase activity. Sections were incubated with rat anti-PNAd antibody (1:25, clone: MECA-79, Biolegend, San Diego, USA, Cat. No. 120801) or mouse anti-FoxP3 antibodies (1:50, clone:235/E7, Abcam, Cambridge, UK, Cat. No. ab20034) at room temperature for 30 minutes or at 4°C overnight. Detection was thereafter carried out by incubating the sections for 30 minutes with HRP-labelled secondary goat anti-rat light chain (1:250, Millipore, Temecula, California, USA, Cat. No AP202P) or anti-mouse IgG (1:100, Sigma‒Aldrich, St. Louis, Missouri, USA, Cat. No A2554) antibodies, followed by diaminobenzidine (Dako EnVision + System-Horseradish Peroxidase, Dako) for visualization. Harris haematoxylin (Sigma‒Aldrich, St. Louis, Missouri, USA) was used for counterstaining. FFPE human lymph nodes or spleen tissue served as positive controls for PNAd and FoxP3, respectively, and for the negative control, the primary antibody was omitted. Assessment of PNAd antibody specificity was performed as described by Wirsing and colleagues [2].

### Immunohistochemical evaluation

The stained sections were scanned with an Olympus VS120 automated slide scanner (Olympus, Germany). For PNAd and CD163, OlyVIA software (version 1.06; Olympus, Germany) was used to digitally capture and evaluate the micrographs, whereas QuPath software (version 0.3.2) [3] was used to evaluate whole-slide Foxp3-stained images.

### PNAd staining for TA-HEVs.

Two trained and calibrated observers independently scored the IHC-stained TA-HEVs. Manual scoring was chosen over automated scoring because HEVs have a unique morphology that requires expert visual judgement for reliable identification and quantification [4,5]. TA-HEVs were defined as distinct brown PNAd staining in aggregates of cells greater than 1. To eliminate HEVs that were not TA, only HEVs that were present within one field of vision of tumour cells were considered. At low-power magnification (5x), five tissue areas with the highest TA-HEVs density (hotspots) were identified for each tissue section, and micrographs were captured at X20 power magnification. The number of TA-HEVs in each hotspot (one visual field at X20 objective) was manually counted, and the mean number per hotspot was calculated for each tumour section as previously described [2]. In situations of disparate scores between the observers, the staining was re-evaluated and discussed to reach agreement. An interrater reliability test was conducted to evaluate the interobserver agreement of the counts.

### CD163 staining for M2 TAMs.

CD163 staining of M2 TAMs revealed cytoplasmic and membrane staining in irregularly shaped cells that overlapped and appeared in clusters, making them difficult to count as individual cells. Thus, the staining was quantified as the mean percentage of positively stained area in five hotspots, each with a magnification of 20X. Quantification was performed using ImageJ/Fiji software (version 2.9.0/1.54d) [6] which can handle overlapping staining and account for the spatial distribution of CD163+ macrophages [7]. Via ImageJ, micrographs of CD163+ hotspots were converted into 8-bit images. For the measurements, the area and area fraction boxes were checked to quantify the micrographs. The threshold was adjusted to ensure that only the positively stained CD163+ cells were visible by setting positive and negative (including background) areas to display contrasting colours. Background staining is a major challenge during the quantification of CD163+ macrophages. For each image to be quantified, background staining was eliminated while ensuring that the total area of interest to be quantified remained unchanged before and after background stain elimination. We subsequently juxtaposed the original unedited CD163+ micrograph with the ImageJ-adjusted micrograph to ensure the best possible elimination of background staining. This approach also aided in identifying an optimal threshold, as the staining intensities of the sections varied somewhat. We used 3 different thresholds, namely, 165, 175 and 150, for weak, optimal, and extremely high staining intensities, respectively, after a consensus among the observers. The quantification results are expressed as the mean percentage of the total area measured in the five hotspots.

### FoxP3 staining for Tregs.

Qupath was selected to evaluate FoxP3 nuclear staining because it possesses advanced cell segmentation algorithms crucial for accurately identifying individual cell nuclei [8]. The automated estimation of stain vectors was performed in a representative image to optimize the digital separation of the haematoxylin and DAB channels and was applied to all images before cell detection. The parameters for positive cell detection were optimized on several images before a threshold level (0.100) was set. Automated positive cell detection was compared with manual counting of positively stained cells in 15 areas corresponding to a 20X field of view (1100x1100 µm; 950331 µm^2^) to determine whether the settings were satisfactory. The threshold was adjusted to 0.150 or 0.175 for a few images with particularly strong staining intensities and/or high background. Scripts for stain vectors and positive cell detection were created to enable batch analysis. Five circular annotations corresponding to 20X field of view were applied to areas with the highest density of FoxP3 cells, referred to as hotspots, in all sections. The number of FoxP3 cells in each of the five hotspots was summed, and the mean number per hotspot was calculated for each section. Each hotspot contained tumour cells and stroma. Areas with high background staining due to red blood cells, blood vessels or tissue artefacts were excluded from annotations to prevent false positive counts.

### Dichotomization Cut-off derivation.

For each patient, the continuous mean marker scores per hot spot were calculated for PNAd, CD163 and FoxP3. Patients were divided into quintiles based on these scores. Cut-off points for dichotomizing scores into low and high were systematically evaluated to optimize separation of cumulative incidence curves for disease-specific death (DSD) using Fine-Gray competing-risk regression models.

The optimal cut-off for each marker was empirically determined at the first quintile (20^th^ percentile) boundary, which maximized separation of cumulative incidence curves:

- PNAd (TA-HEV score): ≤2.20
- CD163 (M2 TAM score): ≤6.339
- FoxP3 (Treg score): ≤72.8

Values at or below these thresholds were classified as “low” and values above these thresholds as “high”. This empirically derived approach ensures internal consistency while explicit reporting of all threshold values enables reproducibility and aligns with REMARK guidelines for transparent biomarker dichotomization [9].

## References

1. Wirsing AM, Rikardsen OG, Steigen SE, Uhlin-Hansen L, Hadler-Olsen E. Characterisation and prognostic value of tertiary lymphoid structures in oral squamous cell carcinoma. BMC Clin Pathol. 2014; 14:38. https://doi.org/10.1186/1472-6890-14-38

2. Wirsing AM, Rikardsen OG, Steigen SE, Uhlin-Hansen L, Hadler-Olsen E. Presence of tumour high-endothelial venules is an independent positive prognostic factor and stratifies patients with advanced-stage oral squamous cell carcinoma. Tumour Biol. 2016; 37:2449–59. https://doi.org/10.1007/s13277-015-4036-4

3. Bankhead P, Loughrey MB, Fernández JA, Dombrowski Y, McArt DG, Dunne PD, et al. QuPath: Open-source software for digital pathology image analysis. Sci Rep. Nature Publishing Group; 2017; 7:16878. https://doi.org/10.1038/s41598-017-17204-5

4. Vella G, Guelfi S, Bergers G. High Endothelial Venules: A Vascular Perspective on Tertiary Lymphoid Structures in Cancer. Front Immunol. 2021; 12:736670. https://doi.org/10.3389/fimmu.2021.736670

5. Sawada J, Hiraoka N, Qi R, Jiang L, Fournier-Goss AE, Yoshida M, et al. Molecular Signature of Tumor-Associated High Endothelial Venules That Can Predict Breast Cancer Survival. Cancer Immunol Res. 2022; 10:468–81. https://doi.org/10.1158/2326-6066.CIR-21-0369

6. Lunde A, Glover JC. A versatile toolbox for semi-automatic cell-by-cell object-based colocalization analysis. Scientific Reports. 2020; 10:19027. https://doi.org/10.1038/s41598-020-75835-7

7. Van Elsas MJ, Labrie C, Etzerodt A, Charoentong P, Van Stigt Thans JJC, Van Hall T, et al. Invasive margin tissue-resident macrophages of high CD163 expression impede responses to T cell-based immunotherapy. J Immunother Cancer. 2023;11: e006433. https://doi.org/10.1136/jitc-2022-006433

8. Mi H, Gong C, Sulam J, Fertig EJ, Szalay AS, Jaffee EM, et al. Digital Pathology Analysis Quantifies Spatial Heterogeneity of CD3, CD4, CD8, CD20, and FoxP3 Immune Markers in Triple-Negative Breast Cancer. Front Physiol. 2020; 11:583333. https://doi.org/10.3389/fphys.2020.583333

9. Sauerbrei W, Taube SE, McShane LM, Cavenagh MM, Altman DG. Reporting Recommendations for Tumor Marker Prognostic Studies (REMARK): An Abridged Explanation and Elaboration. J Natl Cancer Inst. 2018; 110:803–11. https://doi.org/10.1093/jnci/djy088
