# Supplementary material for: FoxP3+ Cells and PNAd+ Tumour-Associated High Endothelial Venules: Synergistic Prognostic Markers in Oral Tongue Squamous Cell Carcinoma
Source: Head Neck Pathol. 2026 Mar 26;20(1):35. doi: 10.1007/s12105-026-01904-4 (PMC13022141; doi:10.1007/s12105-026-01904-4)
Supplement: Supplementary file 2 — Supplementary Material 2 [file 12105_2026_1904_MOESM2_ESM.docx]

# SUPPLEMENTARY DATA

### Manuscript Title: FoxP3+ Cells and PNAd+ Tumour-Associated High Endothelial Venules: Synergistic Prognostic Markers in Oral Tongue Squamous Cell Carcinoma

**This file includes:**

- Supplementary Methods S1 to S4
- Supplementary Results
- Supplementary Figure S1
- Supplementary Tables S1 to S2
- Supplementary References

## SUPPLEMENTARY METHODS

### S1. Sensitivity Analyses for Cut-Off Point Robustness

### Rationale

No universally accepted cut-off values exist for PNAd or FoxP3 in oral tongue squamous cell carcinoma (OTSCC). To ensure observed prognostic associations were robust rather than dependent on data-driven cut-point selection, we performed comprehensive sensitivity analyses using multiple alternative modelling strategies.[1]

### Primary Cut-Point Strategy (Main Analysis)

The primary analysis employed quintile-based dichotomisation at the 20th percentile:

- PNAd: ≤2.20 HEVs per hotspot (Low) vs >2.20 (High reference)

- FoxP3: ≤72.8 cells per hotspot (Low reference) vs >72.8 (High)

This threshold: (1) identifies tumours with distinctly low biomarker density, (2) maintains adequate sample sizes for multivariable modelling, (3) provides clinical interpretability, and (4) aligns with REMARK guidelines.[2,3]

### Alternative Strategies Tested

To assess robustness, we evaluated:

**1. Continuous Variable Models**

- PNAd: HEV density score (continuous)

- FoxP3: Mean cell counts per hotspot, scaled per 50 cells

- Advantage: Maximum statistical power, no information loss [1]

**2. Median Split Models**

- Dichotomisation at the 50th percentile

- Advantage: Balanced group sizes, facilitates cross-study comparison

**3. Tertile Split Models**

- Three categories (33rd and 67th percentiles)

- Reference: Middle tertile

- Advantage: Captures non-linear relationships

### Model Specifications

For each strategy, two model types were evaluated:

- Individual Models: Biomarker as sole immunohistochemical variable, adjusted for age, sex, T-status, N-status, and histological grade

- Combined Models: Both PNAd and FoxP3 included simultaneously with same clinical covariate adjustment

All sensitivity analyses employed Fine–Gray competing-risk regression [4] with robust standard errors (n=117 for PNAd; n=109 for FoxP3).

### Interpretation Framework

Robustness was evaluated based on consistency of effect direction and statistical evidence across continuous and categorical modelling strategies, together with preservation of the context-dependent relationship between PNAd and FoxP3.

### S2. Incremental Prognostic Value Beyond pTNM Stage

### Overview

To determine whether immune biomarkers provided prognostic information beyond established pTNM stage, we compared nested Fine–Gray competing-risk regression models. Analyses were restricted to patients with complete covariate data (n=112). Baseline characteristics did not differ between complete cases and patients with missing FoxP3 (n=8; all p>0.05).

### Model Structure

- **Model 1 (Baseline):** Age, sex, histological grade, pTNM stage

- **Model 2:** Baseline + PNAd

- **Model 3**: Baseline + FoxP3

- **Model 4**: Baseline + PNAd + FoxP3

### Evaluation Metrics

Incremental value assessed using three complementary metrics [5]:

**1. Wald Tests:** Independent associations with disease-specific mortality beyond clinical covariates

**2. AICc (Model Fit):** Corrected Akaike Information Criterion with finite-sample correction [6]

- ΔAICc >2: improved fit

- ΔAICc >6: strong evidence for more complex model

- Formula: AICc = AIC + [2k(k+1)] / (n-k-1)

**3. C-Index (Discrimination):** Harrell's concordance index [7] (see S3)

- ΔC ≥0.05: clinically meaningful improvement

- ΔC ≥0.10: substantial improvement

Related to Table S2 (this file)

### S3. C-Index Estimation in Competing-Risk Settings

### Challenge

Stata's `estat concordance` does not support Fine–Gray competing-risk models (`stcrreg`).

### Solution

We used a validated Cox regression proxy approach to estimate C-indices for Fine–Gray models with identical covariate specifications.

### Methodological Justification

Theoretical Basis:

- C-index measures concordance between predicted risks and observed outcomes

- For disease-specific death, risk rankings should be similar between the Cox and Fine–Gray models when competing risk incidence is moderate

- Wolbers et al. [8] demonstrated that Cox-based C-indices provide reasonable approximations when competing event incidence is <30%

- Our cohort: Competing events = 14/112 = 12.5% ✓

**Implementation:**

- Competing events treated as censored in Cox models (appropriate for C-index estimation only)

- Fine–Gray models retained for all reported hazard ratios

- Proportional hazards assumption tested using time-varying covariates (no violations detected)

**C-Index Interpretation:**

- 0.5 = random prediction

- 0.6–0.7 = modest discrimination

- 0.7–0.8 = acceptable discrimination

- >0.8 = excellent discrimination

### Limitations

- Cox-based C-indices provide approximations, not exact Fine–Gray C-indices

- C-index increases interpreted alongside other metrics (ΔAICc, Wald tests)

- External validation essential before clinical implementation

### S4. Sample Size and Power Considerations

### Complete Case Analysis

**Sample:**

- Total eligible: n=120 patients

- Complete for all variables: n=112 patients

- Excluded: n=8 (missing FoxP3 due to insufficient tissue)

**Outcome Distribution (n=112):**

- Disease-specific deaths: 34 (30.4%)

- Competing deaths: 14 (12.5%)

- Censored: 64 (57.1%)

### Events-Per-Variable (EPV) Ratio

**Model 4 (Combined Biomarker Model):**

- Total parameters: 7 [Stage (2), Grade (1), Age (1), Sex (1), PNAd (1), FoxP3 (1)]

- Disease-specific events: 34

- **EPV = 34 ÷ 7 = 4.9**

**Methodological Concern:**

- Recommended threshold: EPV ≥10 [9]

- Our ratio (4.9) is below this threshold

- Risk: Potential overfitting and optimistic effect size estimates

**Mitigation Strategies:**

1. Finite-sample correction: AICc used (not AIC)

2. Competing-risk methodology: may reduce bias in risk estimation in the presence of competing events

3. Sensitivity analyses: Robustness across multiple cut-point strategies

4. Consistent sample: All nested models on identical n=112

5. External validation: Acknowledged as essential next step

**Interpretation:**

Effect sizes should be interpreted as preliminary estimates requiring validation. Consistent patterns across multiple modelling strategies and metrics strengthen confidence.

### Comparison of Complete vs Incomplete Cases

Baseline characteristics (n=112 vs n=8):

| **Variable** | **Complete (n=112)** | **Missing FoxP3 (n=8)** | **p-value** |
| --- | --- | --- | --- |
| Age ≥65 years | 45.5% | 50.0% | >0.05 |
| Male sex | 60.7% | 62.5% | >0.05 |
| N+ stage | 15.2% | 12.5% | >0.05 |
| High T-stage | 64.3% | 62.5% | >0.05 |
| Poor grade | 12.5% | 12.5% | >0.05 |

**Conclusion:** No significant differences detected, indicating missing FoxP3 data unlikely to introduce systematic bias.

## SUPPLEMENTARY RESULTS

### Incremental Prognostic Value of Immune Biomarkers

To establish whether immune biomarkers provided prognostic value beyond established clinicopathological parameters, we compared nested Fine–Gray competing-risk models (Table S2). The baseline clinical model (Model 1) demonstrated acceptable discrimination (C-index=0.727). The addition of PNAd alone (Model 2) provided minimal improvement over the baseline (ΔAICc=−0.10). Conversely, the addition of FoxP3 alone (Model 3) substantially improved model fit and discrimination (ΔAICc=−5.23, ΔC=+0.043). The combined biomarker approach (Model 4) yielded the best-performing prognostic model (ΔAICc=−8.93, ΔC=+0.057). Notably, PNAd demonstrated context-dependent prognostic utility, reaching statistical significance only when evaluated alongside FoxP3. Collectively, these findings confirm that the combined assessment of FoxP3 and PNAd provides a clinically meaningful improvement in risk discrimination beyond standard pTNM staging.

### Model 1: Baseline Clinical Model

- **Performance:** AICc=300.46, C-index=0.727

- **Findings:**

- Stage III/IV: sHR=5.98 (95% CI 1.71–20.95), p=0.005

- Stage II: sHR=3.67 (95% CI 0.95–14.24), p=0.060

- Poor differentiation: sHR=3.47 (95% CI 1.43–8.44), p=0.006

### Model 2: Baseline + PNAd

- Performance: AICc=300.36 (ΔAICc=−0.10), C-index=0.736 (Δ=+0.009)

- Findings: PNAd not independently associated (sHR=1.86, 95% CI 0.88–3.91, p=0.104)

- Interpretation: Minimal improvement over baseline

### Model 3: Baseline + FoxP3

- **Performance**: AICc=295.23 (ΔAICc=−5.23), C-index=0.770 (Δ=+0.043)

- **Findings:** High FoxP3 independently associated with mortality (sHR=4.95, 95% CI 1.72–14.24, p=0.003)

- **Interpretation:** Substantial improvement in model fit and discrimination

### Model 4: Combined Biomarker Model (Optimal)

- **Performance**: AICc=291.53 (ΔAICc=−8.93), C-index=0.784 (Δ=+0.057)

- **Findings:**

- Low PNAd: sHR=2.86 (95% CI 1.36–6.01), p=0.006

- High FoxP3: sHR=7.78 (95% CI 3.41–17.71), p<0.001

- Joint Wald test: χ²=25.90 (df=2), p<0.0001

- **Interpretation:**

- Evidence of improved prognostic information (ΔAICc>6)

- Clinically meaningful improvement in discrimination (ΔC>0.05)

- PNAd prognostic association emerges when evaluated with FoxP3 (context-dependent)

### Key Findings

1. **FoxP3:** Consistent independent prognostic value across all model configurations

2. **PNAd:** Context-dependent prognostic utility (significant only when combined with FoxP3)

3. **Combined Assessment:** Provides incremental value beyond pTNM staging across all three metrics

4. **C-Index Improvement:** Model 4 exceeds clinically meaningful threshold (Δ=+0.057>0.05)

## SUPPLEMENTARY FIGURES

### Supplementary Figure S1

**Supplementary Figure S1. Spatial proximity of FoxP3+ cells and PNAd+ TA-HEVs in the TME of OTSCC.** Representative spatial relationship between FoxP3+ cells and PNAd+ TA-HEVs in OTSCC tissue. Separate IHC staining for HEVs (left panel) and FOXP3 (right panel) on adjacent sections of the same tumour region.

## SUPPLEMENTARY TABLES

### Supplementary Table S1. Sensitivity Analyses of PNAd and FoxP3 Using Alternative Modelling Strategies

### Panel A: Continuous Variable Models

| **Variable** | **Model** | **sHR** | **95% CI** | **p-value** | **n** | **Events** |
| --- | --- | --- | --- | --- | --- | --- |
| PNAd (HEV_score) | Individual | 0.930 | 0.769–1.123 | 0.450 | 117 | 33 |
| FoxP3 (per 50-cell increase) | Individual | 1.139 | 1.039–1.249 | 0.006 | 109 | 32 |
| PNAd (HEV_score) | Combined | 0.925 | 0.755–1.133 | 0.453 | 109 | 32 |
| FoxP3 (per 50-cell increase) | Combined | 1.145 | 1.044–1.256 | 0.004 | 109 | 32 |

### Panel B: Median Split Models

| **Variable** | **Model** | **sHR** | **95% CI** | **p-value** | **n** | **Events** |
| --- | --- | --- | --- | --- | --- | --- |
| PNAd Low (vs High) | Individual | 1.048 | 0.498–2.206 | 0.901 | 117 | 33 |
| FoxP3 High (vs Low) | Individual | 1.470 | 0.671–3.219 | 0.335 | 109 | 32 |
| PNAd Low (vs High) | Combined | 1.012 | 0.456–2.246 | 0.976 | 109 | 32 |
| FoxP3 High (vs Low) | Combined | 1.474 | 0.644–3.375 | 0.359 | 109 | 32 |

### Panel C: Tertile Split Models

### PNAd Tertiles (Reference: Middle Tertile)

| **Category** | **Model** | **sHR** | **95% CI** | **p-value** | **n** | **Events** |
| --- | --- | --- | --- | --- | --- | --- |
| Tertile 0 (Lowest) | Individual | 1.337 | 0.601–2.975 | 0.477 | 117 | 33 |
| Tertile 2 (Highest) | Individual | 0.984 | 0.385–2.513 | 0.973 | 117 | 33 |
| Tertile 0 (Lowest) | Combined | 1.105 | 0.468–2.613 | 0.820 | 109 | 32 |
| Tertile 2 (Highest) | Combined | 0.985 | 0.399–2.429 | 0.973 | 109 | 32 |

### FoxP3 Tertiles (Reference: Lowest Tertile)

| **Category** | **Model** | **sHR** | **95% CI** | **p-value** | **n** | **Events** |
| --- | --- | --- | --- | --- | --- | --- |
| Tertile 1 (Middle) | Individual | 1.006 | 0.388–2.607 | 0.990 | 109 | 32 |
| Tertile 2 (Highest) | Individual | 2.329 | 0.901–6.024 | 0.081 | 109 | 32 |
| Tertile 1 (Middle) | Combined | 1.035 | 0.390–2.744 | 0.945 | 109 | 32 |
| Tertile 2 (Highest) | Combined | 2.327 | 0.914–5.925 | 0.077 | 109 | 32 |

### Panel D: Summary of Key Findings Across Modelling Strategies

| **Biomarker** | **Continuous** | **Median** | **Tertile** | **Quintile (Main)** | **Interpretation** |
| --- | --- | --- | --- | --- | --- |
| FoxP3 (individual) | p=0.006 | p=0.335 | p=0.081 | p=0.003 | Consistent prognostic value |
| FoxP3 (combined) | p=0.004 | p=0.359 | p=0.077 | p<0.001 | Strengthened with PNAd |
| PNAd (individual) | p=0.450 | p=0.901 | p=0.477/0.973 | p=0.104 | Non-significant alone |
| PNAd (combined) | p=0.453 | p=0.976 | p=0.820/0.973 | p=0.006 | Context-dependent |

**Sensitivity Analyses of PNAd⁺ and FoxP3⁺ Using Alternative Modelling Strategies.** ᵃMain analysis used quintile-based cut-off (20th percentile); see Table 4 and Table 5 in the main manuscript. Subdistribution hazard ratios (sHR) and 95% confidence intervals (CI) were estimated using Fine–Gray competing-risk regression (disease-specific death within 5 years, with non-disease-related death as a competing event). All models were adjusted for age (≥65 years), sex, T-status, N-status, and histological grade. Detailed methodological rationale, variable definitions, and modelling interpretations are provided in Supplementary Methods S1. *Abbreviations:* CI, confidence interval; sHR, subdistribution hazard ratio; TA-HEV, tumor-associated high endothelial venule; vs, versus.

### Supplementary Table S2. Complete Coefficients for Nested Prognostic Models

### Panel A: Complete Model Coefficients

| **Variable** | **Model 1 (Baseline clinical)** | **Model 2 (+ PNAd)** | **Model 3 (+ FoxP3)** | **Model 4 (Combined)** |
| --- | --- | --- | --- | --- |
| Sample Size | n=112 | n=112 | n=112 | n=112 |
| Events (DSD/Competing) | 34/14 | 34/14 | 34/14 | 34/14 |
| **CLINICAL VARIABLES** |  |  |  |  |
| Stage II | 3.67 (0.95–14.24); p=0.060 | 3.67 (0.94–14.37); p=0.062 | 4.35 (1.10–17.11); p=0.035 | 4.15 (1.06–16.29); p=0.041 |
| Stage III | 5.98 (1.71–20.95); p=0.005 | 5.97 (1.67–21.37); p=0.006 | 6.18 (1.74–21.99); p=0.005 | 6.17 (1.69–22.47); p=0.006 |
| Poor differentiation | 3.47 (1.43–8.44); p=0.006 | 3.12 (1.31–7.48); p=0.011 | 4.13 (1.51–11.27); p=0.006 | 4.54 (1.92–10.71); p=0.001 |
| Age ≥65 | 1.12 (0.56–2.24); p=0.757 | 1.11 (0.56–2.21); p=0.766 | 0.97 (0.46–2.01); p=0.926 | 0.84 (0.41–1.72); p=0.631 |
| Male sex | 0.84 (0.41–1.73); p=0.634 | 0.79 (0.38–1.66); p=0.540 | 0.80 (0.40–1.63); p=0.540 | 0.82 (0.41–1.64); p=0.576 |
| **IMMUNE BIOMARKERS** |  |  |  |  |
| PNAd Low | — | 1.86 (0.88–3.91); p=0.104 | — | 2.86 (1.36–6.01); p=0.006 |
| FoxP3 High | — | — | 4.95 (1.72–14.24); p=0.003 | 7.78 (3.41–17.71); p<0.001 |

### Panel B: Model Performance Metrics

| **Metric** | **Model 1** | **Model 2** | **Model 3** | **Model 4** |
| --- | --- | --- | --- | --- |
| Log-likelihood | -144.95 | -143.78 | -141.21 | -138.23 |
| AICc | 300.46 | 300.36 | 295.23 | 291.53 |
| ΔAICc vs Model 1 | Reference | -0.10 | -5.23 | -8.93 |
| C-index | 0.727 | 0.736 | 0.770 | 0.784 |
| ΔC vs Model 1 | Reference | +0.009 | +0.043 | +0.057 |
| Wald χ² (df) | — | 2.65 (1) | 8.81 (1) | 25.90 (2) |
| p-value | — | 0.104 | 0.003 | <0.0001 |

### Panel C: Model Comparison Summary

| **Comparison** | **Added Variable(s)** | **ΔAICc** | **Interpretation** | **Wald Test** | **C-index Improvement** |
| --- | --- | --- | --- | --- | --- |
| M2 vs M1 | PNAd alone | -0.10 | Minimal improvement | p=0.104 | +0.009 |
| M3 vs M1 | FoxP3 alone | -5.23 | Strong improvement | p=0.003 | +0.043 |
| M4 vs M1 | Both biomarkers | -8.93 | Strong improvement | p<0.0001 | +0.057 |

**Complete Coefficients and Performance Metrics for Nested Prognostic Models.** Analysis restricted to complete cases (n=112) to allow for valid AICc comparison. The baseline clinical model includes composite pTNM stage (Stage III/IV merged), histological grade, age, and sex. Detailed parameter definitions, mitigation strategies for EPV <10, and justification for C-index proxy estimation are provided in Supplementary Methods S2–S4. *Abbreviations:* AICc, corrected Akaike Information Criterion; CI, confidence interval; C-index, concordance index; df, degrees of freedom; DSD, disease-specific death; EPV, events per variable; pTNM, pathological tumor-node-metastasis; sHR, subdistribution hazard ratio. *p<0.05; **p<0.01; ***p<0.001.

Collectively, these supplementary analyses demonstrate that the observed prognostic associations were consistent across multiple modelling strategies, supporting the robustness of the findings and indicating that results were unlikely to be driven by cut-point selection or model specification.

## REFERENCES

1. Collins GS, Ogundimu EO, Cook JA, Manach YL, Altman DG. Quantifying the impact of different approaches for handling continuous predictors on the performance of a prognostic model. Stat Med. 2016;35:4124–35. https://doi.org/10.1002/sim.6986

2. Sauerbrei W, Taube SE, McShane LM, Cavenagh MM, Altman DG. Reporting Recommendations for Tumor Marker Prognostic Studies (REMARK): An Abridged Explanation and Elaboration. J Natl Cancer Inst. 2018;110:803–11. https://doi.org/10.1093/jnci/djy088

3. Altman DG, Lausen B, Sauerbrei W, Schumacher M. Dangers of using “optimal” cutpoints in the evaluation of prognostic factors. J Natl Cancer Inst. 1994;86:829–35. https://doi.org/10.1093/jnci/86.11.829

4. Fine JP, Gray RJ. A Proportional Hazards Model for the Subdistribution of a Competing Risk. Journal of the American Statistical Association. ASA Website; 1999;94:496–509. https://doi.org/10.1080/01621459.1999.10474144

5. Pencina MJ, D’ Agostino Sr RB, D’ Agostino Jr RB, Vasan RS. Evaluating the added predictive ability of a new marker: From area under the ROC curve to reclassification and beyond. Statistics in Medicine. 2008;27:157–72. https://doi.org/10.1002/sim.2929

6. Burnham KP, Anderson DR, Anderson DR. Model selection and multimodel inference: a practical information-theoretic approach. 2. ed., [4. printing]. New York, NY: Springer; 2010.

7. Harrell FE, Califf RM, Pryor DB, Lee KL, Rosati RA. Evaluating the yield of medical tests. JAMA. 1982;247:2543–6.

8. Wolbers M, Koller MT, Witteman JCM, Steyerberg EW. Prognostic Models With Competing Risks: Methods and Application to Coronary Risk Prediction. Epidemiology. 2009;20:555. https://doi.org/10.1097/EDE.0b013e3181a39056

9. Vittinghoff E, McCulloch CE. Relaxing the rule of ten events per variable in logistic and Cox regression. Am J Epidemiol. 2007;165:710–8. https://doi.org/10.1093/aje/kwk052
